# Adult Pancreas Side Population Cells Expand after β Cell Injury and Are a Source of Insulin-Secreting Cells

**DOI:** 10.1371/journal.pone.0048977

**Published:** 2012-11-09

**Authors:** Ilia Banakh, Leonel J. Gonez, Robyn M. Sutherland, Gaetano Naselli, Leonard C. Harrison

**Affiliations:** 1 The Walter and Eliza Hall Institute of Medical Research, Parkville, Victoria, Australia; 2 Department of Medical Biology, The University of Melbourne, Parkville, Victoria, Australia; University of Minnesota Medical School, United States of America

## Abstract

Pancreas stem cells are a potential source of insulin-producing β cells for the therapy of diabetes. In adult tissues the ‘side population’ (SP) of cells that effluxes the DNA binding dye Hoechst 33342 through ATP-binding cassette transporters has stem cell properties. We hypothesised therefore that the SP would expand in response to β cell injury and give rise to functional β cells. SP cells were flow sorted from dissociated pancreas cells of adult mice, analysed for phenotype and cultured with growth promoting and differentiation factors before analysis for hormone expression and glucose-stimulated insulin secretion. SP cell number and colony forming potential (CFP) increased significantly in models of type diabetes, and after partial pancreatectomy, in the absence of hyperglycaemia. SP cells, ∼1% of total pancreas cells at 1 week of age, were enriched >10-fold for CFP compared to non-SP cells. Freshly isolated SP cells contained no insulin protein or RNA but expressed the homeobox transcription factor Pdx1 required for pancreas development and β cell function. Pdx1, along with surface expression of CD326 (Ep-Cam), was a marker of the colony forming and proliferation potential of SP cells. In serum-free medium with defined factors, SP cells proliferated and differentiated into islet hormone-expressing cells that secreted insulin in response to glucose. Insulin expression was maintained when tissue was transplanted within vascularised chambers into diabetic mice. SP cells in the adult pancreas expand in response to β cell injury and are a source of β cell progenitors with potential for the treatment of diabetes.

## Introduction

Loss of insulin production in type 1 diabetes (T1D) following autoimmune destruction of β cells in the islets of the pancreas requires life-long treatment with insulin injections. Insulin treatment is demanding and rarely achieves physiologic control of blood glucose. Transplantation of the pancreas or pancreatic islets can restore near-normal glucose homeostasis, but is technically demanding, expensive and limited by a shortage of donor tissue, and not all recipients achieve insulin-independence [1], [2]. Thus, there is a strong imperative to derive renewable sources of insulin-producing cells to ‘cure’ T1D. Defined growth-differentiation conditions have been used to coax development of embryonic stem cells (ESCs) through definitive endoderm and pancreatic endoderm to insulin-producing endocrine cells [3], [4]. However, scale-up has proved challenging and is yet to yield sufficient cells capable of reversing hyperglycaemia in large animal models. In addition, chromosomal instability in ESCs and their potential for oncogenesis *in vivo* remain a concern [5], [6].

Stem or progenitor cells in the adult pancreas are potentially an alternative source of β cells [7], [8]. Evidence for attempted β cell regeneration, in the face of ongoing autoimmunity, has been noted in humans with established T1D [9], [10]. Additionally, pancreatic duct replication was identified in human T2D [11]. More compelling is the evidence for β cell regeneration from duct-associated stem/progenitor cells [12]–[14] or β cell self-duplication [15], [16] in rodent models of β cell or pancreas injury. Injury induced by pancreatic duct ligation in the mouse resulted in the accumulation of cells expressing the endocrine progenitor marker Ngn3 in newly formed duct complexes [12], which when transplanted into *Ngn3−/−* pancreatic primordia *in vitro* gave rise to islet endocrine cells [12]. Duct-derived cells traced by the lineage marker, carbonic anhydrase II, were shown to give rise to both islet and acinar cells after pancreatic duct ligation [13]. In a further example, in response to partial pancreatectomy rat duct cells proliferated and replicated stages of embryonic development to form pancreatic lobes [14].

**Table 1:** Antibodies used to stain the mouse pancreas side population.

Antibody	Nomenclature	Dilution	Source
Rat anti-mouse Ly-5	CD45	1∶10**^3^**	BD
Rat anti-mouse c-kit	CD117	1∶200	BD
Rat anti-mouse Thy-1	CD90	1∶200	Labvision
Rat anti-mouse CD24	CD24	1∶300	BD
Rat anti-mouse DPP IV	CD26	1∶200	BD
Hamster anti- human integrin β1	CD29	1∶400	Cymbus
Mouse anti-human integrins α6	CD49f	1∶300	BD
Rat anti-mouse Sca-1	–	1∶300	BD
Rat anti-mouse prominin	CD133	1∶300	eBioscience
Rat anti-mouse Ep-CAM	CD326	1∶400	BD
Dolichos biflorus agglutinin (lectin)	–	1∶500	Vector

Endocrine progenitors from mouse pancreas [17], [18], human pancreatic ducts [19] and human foetal pancreas [20] have been isolated using cell surface marker antibodies. However, these markers are not entirely specific for stem-progenitor cells because of cellular heterogeneity and lineage marker overlap at different stages of development [21]. Putative stem cells have also been identified by the capacity to efflux the DNA binding dye Hoechst 33342 through surface ATP binding cassette (ABC) transporters [22]. The latter include breast cancer resistance protein 1 (BCRP1, also known as ATP-binding cassette sub-family G member 2 [ABCG2]) and multidrug resistance protein 1 (MDR1, also known as ABCB1) [23]. By flow cytometry, cells that efflux Hoechst 33342 are detected as a low fluorescence ‘side population’ (SP). SP cells from the bone marrow have an undifferentiated phenotype, proliferate and exhibit colony forming potential (CFP), and are enriched for long-term, re-populating hematopoietic stem cells [24]. SP cells have also been identified in non-hematopoietic tissues including liver, brain, kidney, lung, skeletal muscle, mammary gland and testis [24], skin [25], intestine [26], prostate [27] and human foetal [28] and adult [29], [30] pancreas, as well as tumor tissues [31], [32]. However, several factors have limited further characterisation and application of SP cells. These include lack of definitive ‘stemness’ markers, low recovery, heterogeneity and inconsistent evidence for regenerative potential. As a test for ‘stemness’, we hypothesised that SP cells in the adult pancreas would increase in frequency and function after β cell or pancreatic injury. We examined this hypothesis to determine if SP cells could be induced to become endocrine cells *in vitro* and maintain insulin expression *in vivo*. We report that SP cells in the adult mouse pancreas expand *in vivo* in response to β cell or pancreas injury and can be induced to proliferate and differentiate *in vitro* to endocrine cells that secrete insulin in response to glucose.

## Materials and Methods

### Animals

Initially, C57BL/6 mice of either sex at 4–8 weeks of age were used to characterize pancreas SP cells. Subsequently, SP cells were isolated from PDX1^GFP/W^ (Pdx1-GFP) mice in which the green fluorescent protein (GFP) gene was targeted to the PDX1 (pancreatic and duodenal homeobox) locus on the 129/SV background, and subsequently backcrossed onto the C57BL/6 background [33]. Four different mouse models of diabetes were used for analysis of the SP response to β cell damage: 1) RIP-H2K^b^ mice that develop non-immune β cell destruction and diabetes by 6 weeks of age due to transgenic overexpression of the MHC class I (K^b^) protein in β cells [34]; 2) Pdx1-GFP mice injected i.p. on 3 consecutive days with the β cell toxin streptozotocin (STZ) (50 mg/kg), leading to diabetes within 14 days; 3) transgenic RIP-CD80-NOD/SCID mice that express the T-cell co-stimulatory CD80 molecule on βcells under control of the rat insulin promoter (RIP) and develop autoimmune diabetes within 3 weeks after transfer of splenic lymphocytes from recently diabetic, non-obese diabetic (NOD) mice [35]; 4) Pdx1-tTA mice express the tetracycline transactivator gene under the control of the Pdx1 promoter and develop diabetes within 2 weeks after doxycycline administration (i.p. injection [1 mg/kg], followed by supplementation in drinking water [0.5 g/L]) [36]. Diabetes was confirmed by a blood glucose concentration persistently >15 mmol/l. After the onset of diabetes, mice were euthanased within 5 days and the pancreas removed for analysis. For partial pancreatectomy in Pdx1-GFP mice, the spleen and the entire splenic portion of the pancreas were surgically removed resulting in 50–60% pancreatectomy. After 1, 2 and 3 weeks, mice were euthanased and remaining pancreas removed for analysis. Transplant recipient mice, the NOD.SCID/IL-2Rgamma null strain, were from the Institute’s animal facility. Experimental procedures were approved by the Animal Ethics Committee of The Walter and Eliza Hall Institute of Medical Research (Ethics permit # 2009.024), undertaken or supervised by trained animal technicians and performed so as to minimize any discomfort to the mice.

**Table 2:** Growth factor combinations.

Compound	Final concentration	Proliferation medium	Differentiation medium
B27 supplement	1x	Yes	Yes
rmEGF	10 µg/L	Yes	Yes
rFGF2	5 µg/L	Yes	No
rFGF10	10 µg/L	Yes	No
Jagged	10 µg/L	Yes	No
LIF	2,000 U/L	Yes	No
ZnSO_4_	10 µM	Yes	Yes
Nicotinamide	12 mM	No	Yes
Betacellulin	10 µg/L	No	Yes
GLP-1	10 µg/L	No	Yes
Retinoic acid	100 nM	No	Yes

### Dissociation of Pancreas Cells

Dissected pancreas was minced in pre-warmed (37°C) red blood cell lysis buffer, washed and digested in ice-cold Hank’s balanced salt solution containing 2 g/L collagenase type III (Worthington Biochemical, Lakewood, NJ, USA), 0.2 g/L soybean trypsin inhibitor (Sigma, Castle Hill, NSW) and 5 g/L deoxyribonuclease I (Sigma) for 20 min at 37°C. The washed digest was incubated with 4 ml of 0.5 g/L trypsin solution (Sigma) for 5 min, blocked by addition of DMEM/F12 medium (Invitrogen) containing 10% foetal calf serum (FCS) (Thermo Corporation, Melbourne, Australia) and cell aggregates were dispersed by pipetting and filtration through a 40 µm nylon strainer.

**Figure 1:**
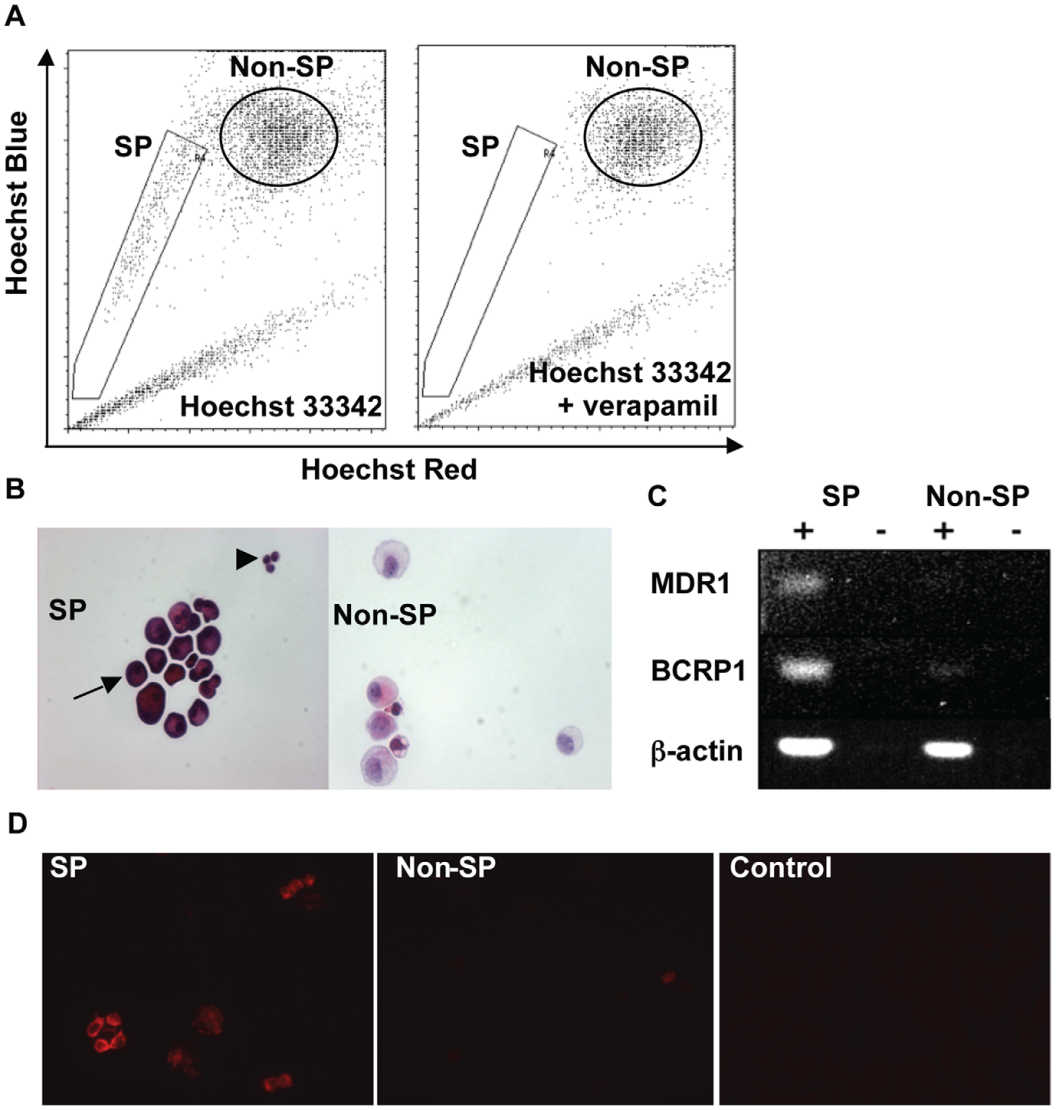
SP cells have distinct morphology and express ABC transporters. A) SP cells and non-SP cells were isolated by FACS after Hoechst 33342 staining. The SP was not detected in the presence of verapamil. B) Hematoxylin and eosin staining of SP and non-SP cells. The majority of SP cells (arrow) had abundant, strongly eosinophilic cytoplasm; a minority were small with little cytoplasmic space (arrowhead). C) Total RNA from cells was analysed for the expression of MDR1 (ABCB1 transporter), BCRP1 (ABCG2 transporter) and β-actin by RT-PCR. D) Immunofluorescence staining of SP and non-SP cells with rat anti-mouse ABCG2 monoclonal antibody. The control was staining with goat anti-rat Ig-PE.

### Cell Labeling with Hoechst 33342 Dye

Pancreas single cell suspensions (10^6^ cells/ml) were incubated in DMEM/F12/5% FCS containing 4 mg/L Hoechst 33342 dye (Invitrogen) at 37°C for 90 min. To inhibit dye efflux, 100 µM verapamil (Sigma) was added to a parallel control sample. To identify and thus exclude hematopoietic cells, phycoerythin-Cy5 (PeCy5)-labelled rat anti-mouse CD45 antibody (BD) was added at a 1∶10^3^ dilution for the final 15 min of the staining. Cell suspensions were chilled for all subsequent steps. Cells were washed and resuspended in cold DMEM/F12 medium containing 2 g/L propidium iodide (PI) to identify and exclude dead cells. Fluorescence activated cell sorting (FACS) was performed on a triple laser FACS-Vantage SE-Diva (BD) instrument using a 351–364 nm argon laser to excite Hoechst 33342 and PI. Emissions were detected at 424/44 nm (Hoechst blue) and 670/40 nm (Hoechst red). CD45-positive cells were excluded from the SP analysis and sorting.

### Cell Labeling and Staining

SP cells were analysed by flow cytometry for hematopoietic and epithelial stem cell surface markers (Table 1). Staining was performed concurrently with that for CD45 during the final 15 min of the Hoechst 33342 labelling. For BrdU labeling in culture sorted cells were incubated for 72 h with BrdU (10 µM, Sigma). Sorted cells were also used for RNA isolation and centrifuged onto coated slides for histology. 10^4^ isolated SP or non-SP cells on slides were fixed in cold methanol for 5 min and stained with hematoxylin and eosin. Digital images were captured with an Axiocam camera from an Olympus BX-50 microscope.

10^4^ cells on slides were fixed in 4% paraformaldehyde, washed in PBS and blocked with 3% normal serum of the secondary antibody host species. Cells were stained with guinea pig anti-swine insulin immunoglobulin (Ig) (1∶400, DAKO) or rat anti-mouse BCRP1 Ig (1∶40, Sapphire Bioscience, Waterloo, NSW) overnight at 4°C. Following washes in PBS, secondary antibodies were added for 2 h at room temperature. Cryosections of adult mouse pancreas were fixed in cold acetone. Non-specific binding was blocked and sections incubated with either rat anti-mouse CD133 Ig (1∶200), rat anti-mouse CD326 Ig (1∶400) or biotinylated DBA lectin (1∶600) for 2 h at room temperature, followed by washes in PBS and 2 h incubation with goat anti-rat Ig-PE (1∶300) or streptavidin-PE (1∶500).

Cells cultured on Matrigel were fixed in 4% Bouins solution for 1 minute and washed in PBS. Autofluorescence was reduced with 0.13 M sodium tetraborate (15 min). Unmasking solution (Vector Laboratories, Burlingame, CA) was used for antigen retrieval, followed by blocking with 3% normal goat serum in PBS. Insulin expression was detected by staining with guinea pig anti-swine insulin Ig (1∶400) followed by FITC-labelled goat anti-guinea pig Ig. To demonstrate the specificity of insulin staining, anti-insulin Ig was pre-incubated for 1 h at room temperature with a combination of human insulin (Novo Nordisk) and proinsulin (produced in-house) (each 20 mg/L). Staining for insulin and BrdU (mouse anti-BrdU Ig 1∶20, BD) were combined, for 2 h at room temperature, followed by washes in PBS and a 2 h incubation with goat anti-guinea pig Ig-FITC (1∶400, Invitrogen) and goat-anti-mouse Ig-Alexa594 (1∶400, Invitrogen). Transplanted tissue was fixed in 4% paraformaldehyde and stained for insulin as described. Immunofluorescent images were captured with an Axiocam camera from a Zeiss Axioplan2 compound microscope.

**Figure 2:**
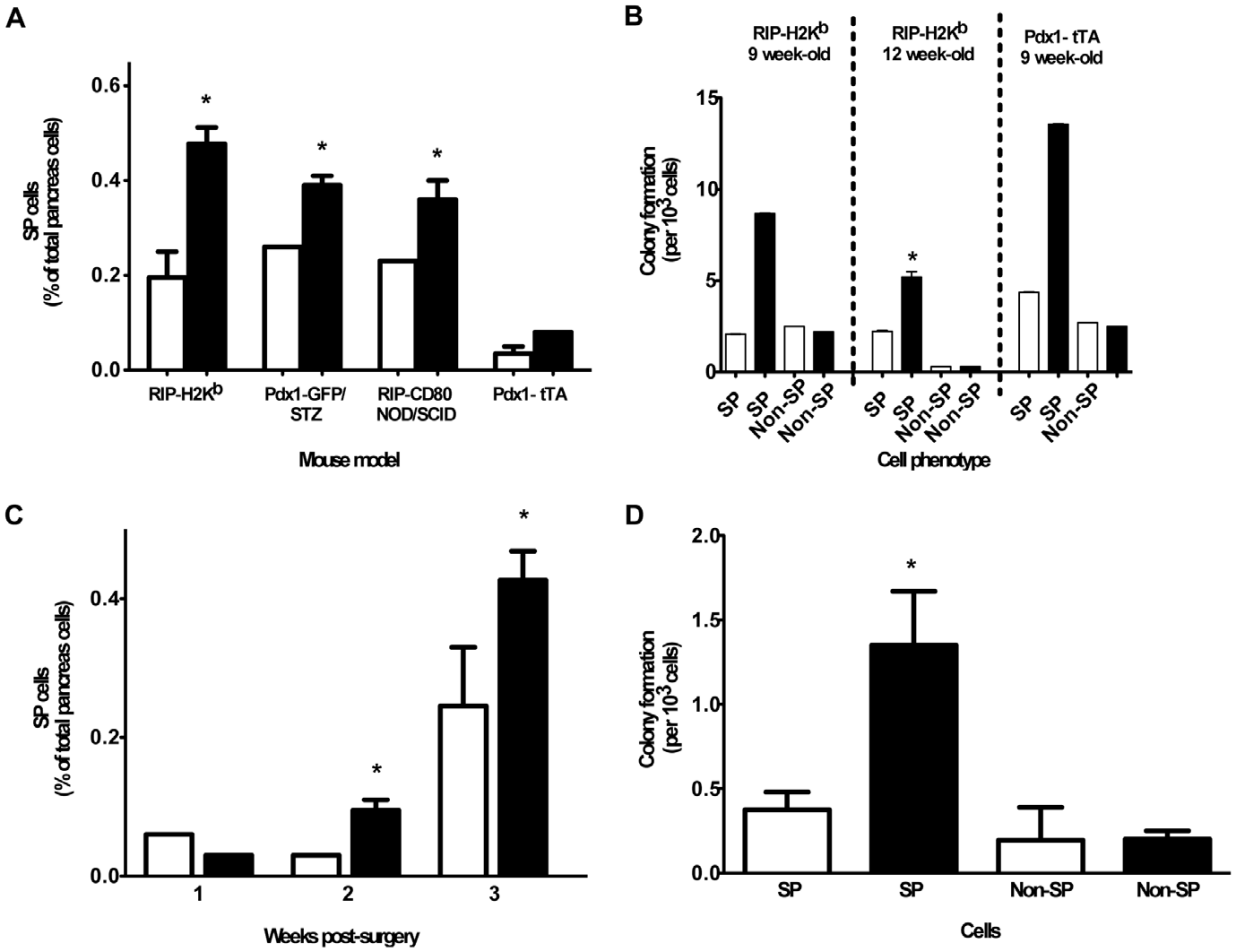
SP cells expand after β cell damage or partial pancreatectomy. A) The SP in four mouse models of β cell damage and diabetes, and B) colony formation in two of these models, were quantified (non-diabetic mice □, diabetic mice ▪, n = 4 mice/group. * p = 0.05). C) Proportion of SP cells and D) colony formation by SP and non-SP cells were quantified in Pdx1-GFP mice starting 2 weeks after splenectomy (□ ) or splenectomy + partial pancreatectomy (▪ ) (n = 4 mice/group. * p = 0.05).

### RNA Extraction and RT-PCR

RNA was extracted with an RNeasy Plus kit (Qiagen) and samples were treated with RNase-free DNase (Qiagen) prior to elution. RNA was reverse transcribed using Superscript First-Strand System (Invitrogen), 0.5 µM random hexanucleotides (Geneworks, Hindmarsh, SA) and 200 µM dNTPs. One-tenth volumes of the first strand synthesis reactions were amplified in PCR buffer (Perkin Elmer, Shelton, CT) containing 200 µM dNTPs, 1 U *Taq* polymerase and 1 µM each of the following gene specific sense/anti-sense oligonucleotide primers: 5′-ggcttccgggaaactcgtgt-3′ and 5′-aagaggtaacatagactggct-3′ annealing at 54°C for BCRP1, 5′-gctcgagcgcttctacgacc-3′ and 5′ggtgcggccttccctggctttg-3′ annealing at 65°C for MDR1, 5′-ggcggccaccctgaacaatg-3′ and 5′-gcccaactccctctccaccaaact-3′ annealing at 58°C for PDX1, 5′ -ccacccaggcttttgtca-3′ and 5′-acaatgccacgcttctgc-3′ annealing at 56°C for insulin, 5′-agggcacattcaccagcgactac-3′ and 5′-cctgcggccgagttcct-3′ annealing at 56°C for glucagon, 5′-gtcctggctttgggcggtgtca-3′ and 5′-cgggggccaggagttaaggaagag-3′ annealing at 66°C for somatostatin, 5′-tgtatgcctcttggtcgtacc-3′ and 5′-caacgtcacacttcatgatgg-3′ annealing at 58°C for β-actin. PCR reactions were preceded by denaturation at 94°C for 3 min. Amplification was for 35 cycles (94°C/15 sec, at annealing temperatures indicated for each primer pair and extension at 72°C/10 sec). Amplified cDNA was analysed by electrophoresis in 1.0% agarose gels.

### Cell Culture

Sorted cells (3×10^3^/well) were maintained in flat-bottom 96-well plates (BD) or 16-well chambered slides (Lab-Tek, Rochester, NY) coated with Matrigel (BD) in serum-free DMEM/F12 (1∶1) medium, as described [37] for chemically defined medium (CDM), with 0.1% w/v polyvinyl alcohol (Sigma P-8136) (P-CDM) replacing BSA. SP cells were propagated through two stages: 1) ‘Proliferation’ for one week (week 1) and 2) ‘Differentiation’ for 2 weeks (weeks 2 and 3). Growth factor and other supplement combinations are listed in Table 2. Medium, changed every second day, was supplemented with Glutamax-1 (2 mM; Invitrogen), soybean trypsin inhibitor (0.1 g/L; Sigma), ITS supplement (insulin [1 g/L], transferrin [0.5 g/L], sodium selenite [0.67 mg/L]; Invitrogen), dexamethasone (10 µM; Sigma), antibiotics (50,000 U penicillin/50 mg streptomycin/L) and B27 supplement (Invitrogen).

**Figure 3:**
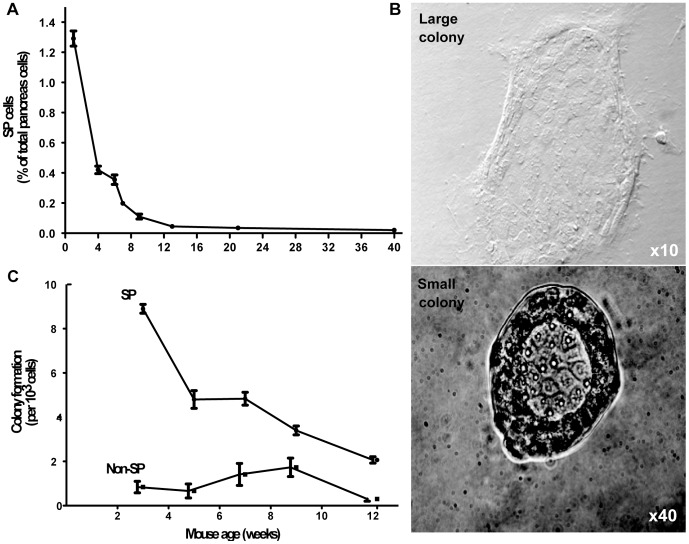
The proportion of SP cells and their colony forming potential decreases with age. A) Pancreas cells from littermates aged 5 days to 40 weeks were stained with Hoechst 33342 dye to quantitate the SP by flow cytometry (n = 7–15 per group). B) Colonies formed by SP and non-SP cells isolated from pancreas of littermates aged 3 to 12 weeks were quantified (n = 7–10 per group). C) Two colony types were observed after 10 days of culture on Matrigel.

**Figure 4:**
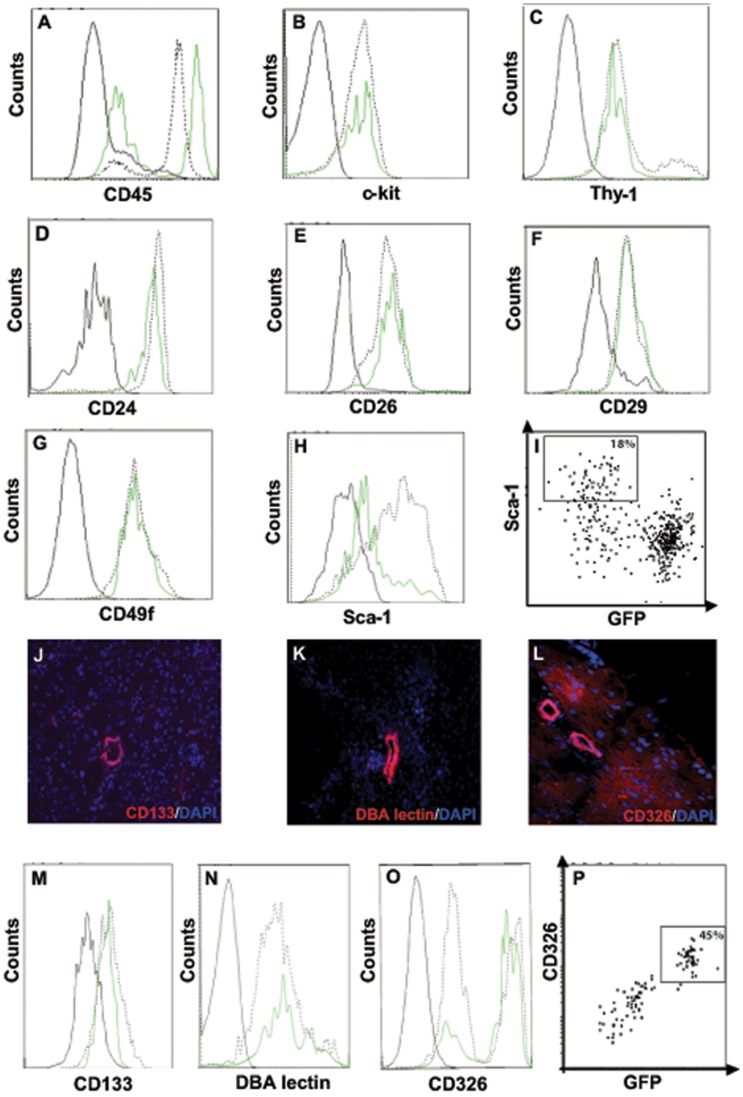
SP and non-SP cells are not distinguished by markers of stem cells or pancreatic duct antigens. A) Expression of CD45 was compared for SP (green line) and non-SP (black dotted line) cells. (B–H) Expression of c-kit, Thy1, CD24, CD26, CD29, CD49f, Sca-1 by CD45^−^ SP cells. The solid black line shows staining by isotype control antibodies. (I) Expression of Sca-1 was analysed in CD45^−^ SP cells from Pdx1-GFP^+^ pancreas. (J–L) Expression of CD133, DBA lectin and CD326 in adult mouse pancreas was confirmed by immunofluoresence staining. (M–O) Expression of CD133, DBA lectin and CD326 by CD45^−^ SP cells. The solid black line shows staining by isotype control antibodies, except for DBA lectin where it represents staining by streptavidin-PE only. (P) Expression of CD326 was analysed in CD45^−^ SP cells from Pdx1-GFP^+^ pancreas.

### Colony Forming Potential

Colony formation was measured by counting colony numbers in primary cultures of sorted cells at the end of the ‘Proliferation’ stage. Colonies had to contain at least 20 cells. Results were expressed in relation to total cells plated.

**Figure 5:**
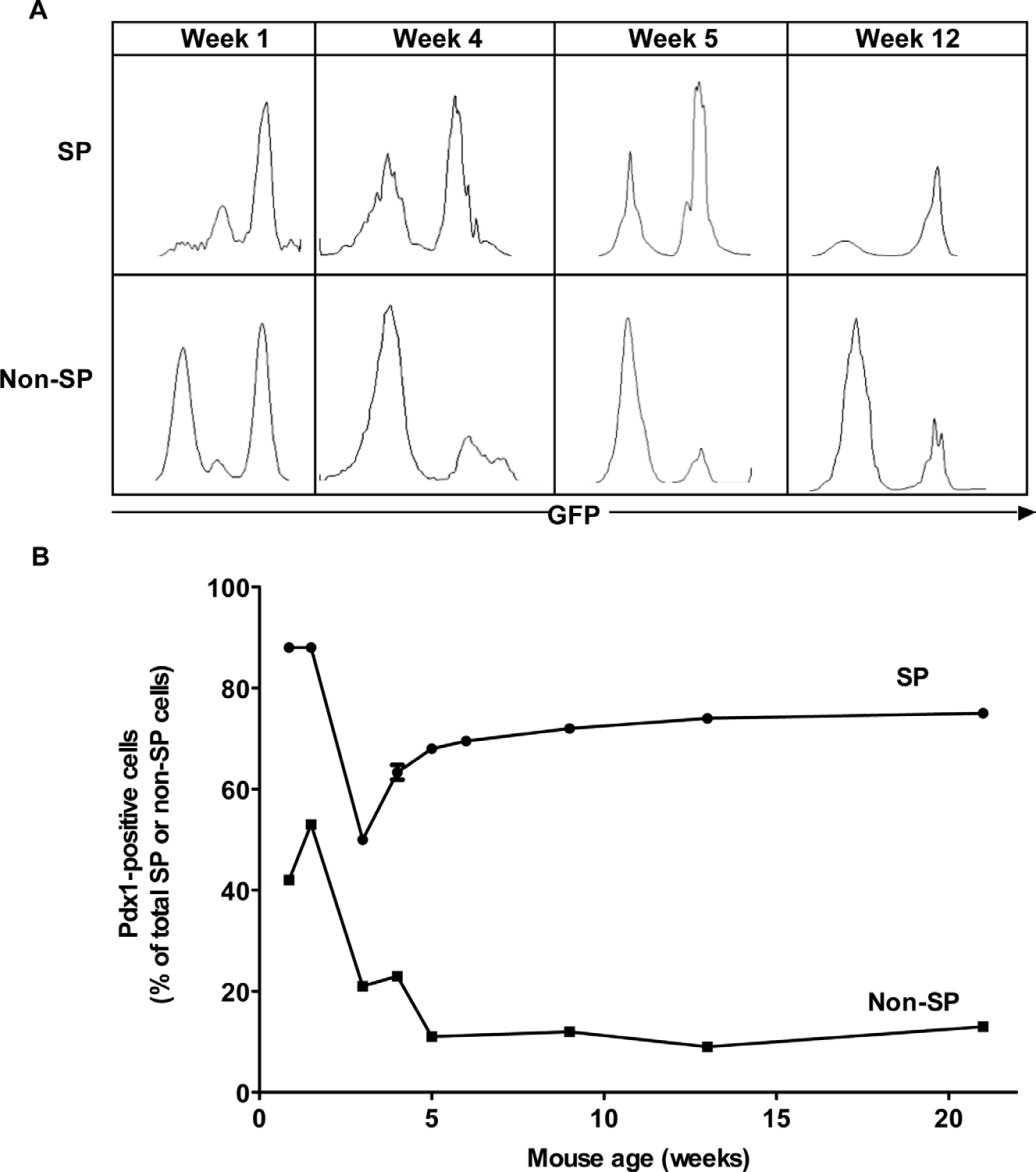
Pdx1 exhibits markedly different expression patterns in SP and non-SP cells. A) Pdx1 expression in SP and non-SP cells from 1–12 week-old Pdx1-GFP mice was measured by flow cytometry. B) Data obtained as in (A) were plotted to demonstrate Pdx1-GFP^+^ expression in SP and non-SP cells from 1–21 weeks of age.

### Glucose-stimulated Insulin Secretion

SP cultures at the end of the ‘Differentiation’ stage were incubated overnight in P-CDM containing no insulin or supplements, washed with the same medium and incubated for 3 h in medium containing 1 mM glucose. Medium was collected, cultures were washed and incubated with medium containing 20 mM glucose for a further 3 h, and medium again collected. Insulin concentration in media was assayed by Immulite 2000 (Siemens Healthcare Diagnostics, USA) using rat insulin as the standard.

### Transplantation into Vascularised Chambers

NOD.SCID/IL-2Rgamma null mice in which diabetes had been induced with streptozotocin were anaesthetised with methoxyflurane. A plastic chamber was implanted around the femoral artery and vein below the groin of each mouse, as described [38]. The chamber was filled with medium containing rhVEGF (R&D Systems) to promote vascularisation, and sealed with bone wax. Four weeks later, cultured SP cell-derived colonies were dislodged with dispase, resuspended in 10 µl of University of Wisconsin solution (gift from St. Vincent’s Research Insitute) and injected transcutaneously into the chamber. Two weeks later, the chamber contents were removed and processed for histological analysis.

**Figure 6:**
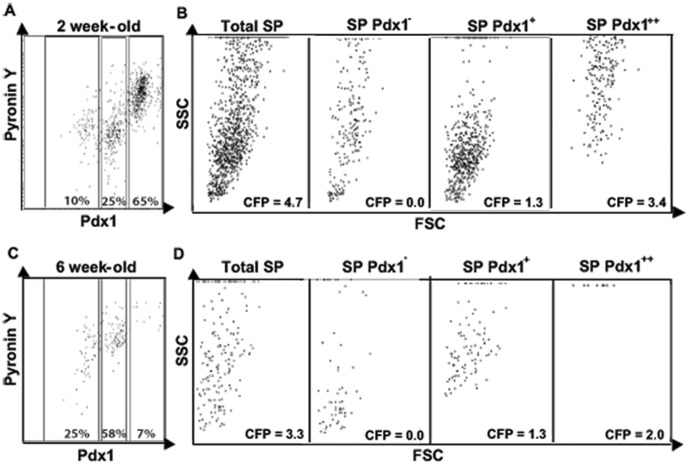
Pdx1 expression reflects proliferation and CFP of SP cells. A,C) Pancreas cells from Pdx1-GFP mice were stained with Hoechst 33342 and then Pyronin Y and analysed by flow cytometry. B,D) Each Pdx1-GFP subpopulation was analysed for size (FSC) and intracellular complexity (SSC). Colony formation in each subpopulation was quantified in 10-day cultures.

### Statistical Methods

Data are presented as mean ± standard error of the mean (SEM). The significance of differences between groups was determined by non-parametric Mann-Whitney tests (two-sided, except in the case of insulin secretion). Statistical analyses were performed with GraphPad Prism v5.0.

## Results

### The SP Expands after β Cell Damage or Partial Pancreatectomy

After staining mouse pancreas cells with Hoechst 33342 dye the SP appeared as a characteristic ‘tail’ from the main (non-SP) population and was not visible in the presence of verapamil (Figure 1A). After excluding CD45-positive cells, the majority of SP cells had intense eosin staining of the cytoplasm; a minority (<10%) were small cells with a higher nucleus to cytoplasm (N/C) ratio (Figure 1B). Non-SP cells comprised mainly larger cells with weak eosin staining and a low N/C ratio (Figure 1B). SP expression of ABC transporters was confirmed by RT-PCR of total RNA from sorted SP and non-SP cells (Figure 1C). Furthermore, immunofluorescence staining revealed that SP cells expressed BCRP1 (Figure 1D).

If SP cells have the capacity to develop into insulin-secreting cells we hypothesised that they would expand in response to β cell or pancreatic injury. Therefore, we quantified the SP in four models of diabetes, as well as in response to partial pancreatectomy. The latter is insufficient to cause diabetes, thus excluding the possibility that an effect is due to hyperglycaemia. One week after the onset of diabetes (3 weeks in RIP-H2K^b^ mice, probably due to the slower development of β cell damage) the SP increased 1.5–2.5-fold (depending on the model) compared with non-diabetic littermate controls (Figure 2A). CFP in the SP, quantified in recently diabetic RIP-H2K^b^ and Pdx1-tTA mice, increased 4.2 and 3.2-fold, respectively; CFP in the non-SP did not change (Figure 2B). After partial pancreatectomy, blood glucose concentration remained less than 12 mM in all mice. Two weeks after pancreatectomy the SP (Figure 2C) and CFP in the SP (Figure 2D) had each increased 3.7-fold compared with splenectomy-only controls. As revealed in Pdx1-GFP mice, the majority of SP cells after pancreatectomy were Pdx1^+^ (data not shown). It should be noted that the mice analysed 3 weeks after pancreatectomy had a higher proportion of SP cells at baseline. These mice were 2 weeks younger than those studied 1 and 2 weeks after pancreatectomy, which lead us to first investigate the relationship between SP cells and age.

**Figure 7:**
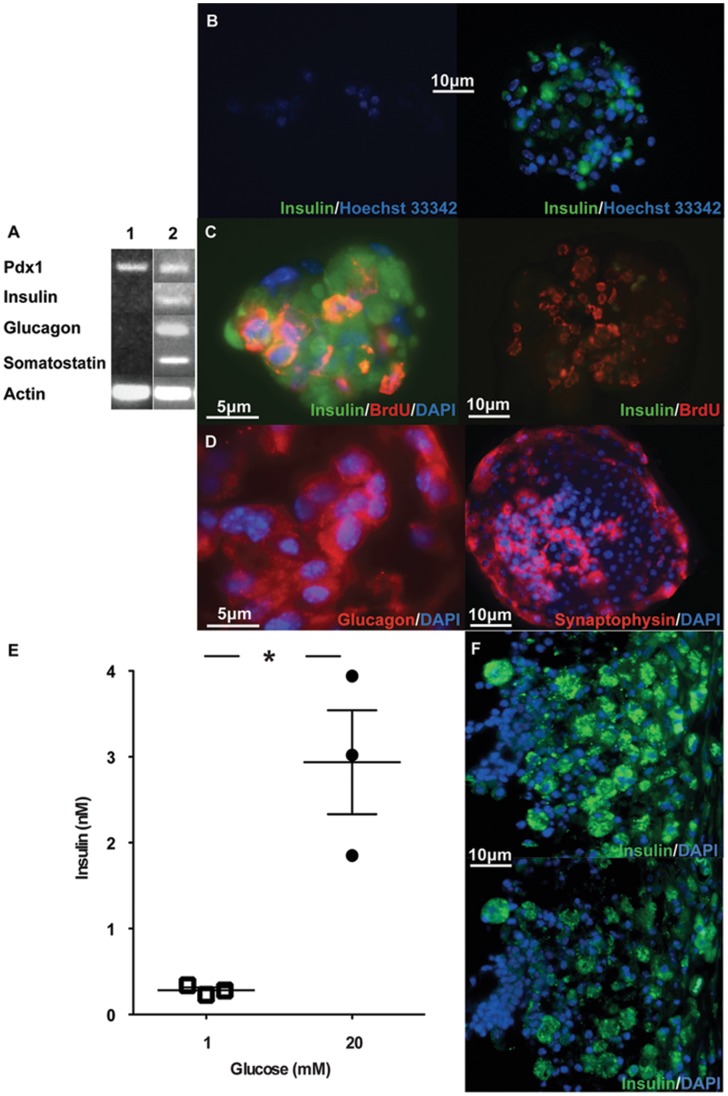
SP cells proliferate and express islet hormones in defined culture conditions. A) Total RNA isolated from fresh SP cells (lane 1) and from SP cells cultured for 3 weeks (lane 2) was analysed by RT-PCR for *PDX1, INSULIN, GLUCAGON, SOMATOSTATIN* and *β-ACTIN* expression. B) SP and non-SP cells were isolated by FACS, cytospun and stained for insulin expression. SP cells had the unique phenotype of low nuclear staining by Hoechst 33342 dye and were negative for insulin expression (left panel). In contrast, non-SP cells had high nuclear staining by Hoechst 33342 and included insulin-positive cells (right panel). C) SP cells were cultured on Matrigel-coated slides in serum-free conditions for 3 weeks, with BrdU for the first 72 h. Growth factors present for the first week were replaced with differentiation factors for the subsequent 2 weeks (see Methods). BrdU uptake into proliferating cells and proinsulin/insulin expression in differentiated cells were identified by indirect immunofluorescence (left panel). Proinsulin/insulin was not detected when the anti-insulin antibody was pre-incubated with a mixture of human proinsulin + insulin (10 mg/L each) (right panel). D) SP cells were cultured on Matrigel-coated slides in serum-free conditions with defined factors for 3 weeks. Indirect immunofluorescence staining for glucagon (left panel) and the secretory vesicle protein, synaptophysin (right panel). E) Colonies derived from 5×10^3^ SP cells cultured in triplicate wells in growth-differentiation conditions for 3 weeks were washed then incubated with 1 mM glucose for 3 h followed by 20 mM glucose for 3 h. Media collected in each period were assayed for insulin and the results compared by paired t test (*p = 0.02). F) Colonies derived from 3-week SP cell cultures in growth-differentiation conditions were transplanted inside vascularised chambers (10^4^ cells per chamber) in NOD/SCID/IL-2rγnull mice rendered diabetic by streptozotocin injection. Chamber contents were recovered 2 weeks later and stained by indirect immunofluorescence for proinsulin/insulin in the absence (top panel) or presence (bottom panel) of human proinsulin + insulin.

### SP Cell Number and Colony Forming Potential Decrease with Age

In 1 week-old C57BL/6 mice, SP cells represented 1.29% ±0.12 of total viable pancreas cells and this proportion rapidly decreased 30-fold in the first 12 weeks of life (Figure 3A). From the neonatal period to 40 weeks of age the proportion of SP cells decreased 65-fold. Ten days after seeding SP cells onto Matrigel-coated plates, two types of colonies were observed, related to the distribution of Matrigel coating. Large, spreading colonies were present centrally in well-gelled areas and smaller, more compact colonies occupied the periphery (Figure 3B). Large colonies grew outwards to cover most of the available surface. Small colonies appeared to be self-limited in the periphery, but then proliferated to fill the inner space. When the inner space was full, branches of cells were observed growing from the luminal area and projecting upwards. These features were not observed in the large colonies. SP cells from weanling mice had >10-fold more colony-forming potential (CFP) than non-SP cells; similar to SP cell number, CFP decreased with age (Figure 3C).

### SP Cells are not Characterised by Markers of Putative Stem Cells

The hematopoietic marker CD45 was expressed by approximately half the pancreatic SP cells (Figure 4A), but unlike CD45^−^ SP cells they did not express Pdx1 or form colonies in culture (see below). These cells of non-pancreatic origin were routinely gated out before flow sorting CD45^−^ SP and non-SP cells. Expression of c-kit, Thy-1, CD24, the incretin-degrading dipeptidyl peptidase CD26 or integrins CD29 and CD49f did not distinguish SP from non-SP cells (Figure 4B–G). Expression of the stem cell marker Sca-1 was higher on non-SP (Figure 4H) and Pdx1-negative, non-colony forming SP cells (Figure 4I). Pancreatic duct epithelium is considered a source of progenitor cells [8], [13] and the duct markers CD133 (prominin), CD326 (Ep-Cam) and *Dolichos biflorus agglutinin* (DBA) lectin (Figure 4J–L) are expressed on putative stem cells isolated from the pancreas and other tissues [39]–[42]. Expression of CD133 and DBA lectin was similar on SP and non-SP cells (Figure 4M and 4N); however, a majority of SP cells compared to less than half the non-SP cells was strongly positive for CD326 (Figure 4O). Moreover, in contrast to Sca-1 (Figure 4I), CD326 identified SP cells that were Pdx1-positive (Figure 4P). Compared to the total SP, colony formation by CD326^+^ SP cells was significantly increased (7.1±2.7 versus 1.9±0.6 colonies/10^3^ cells; p = 0.03). The association between CD326 and Pdx1 expression in SP cells was maintained in pancreatectomised Pdx1-GFP mice.

### SP Cells are Characterised by High Expression of Pdx1, Mirroring their Proliferative and Colony Forming Potential

The homeobox transcription factor Pdx1 is essential for pancreas growth and β cell differentiation and, in the adult pancreas, for maintenance of β cell function and β cell regeneration following pancreas injury [7], [43]. To monitor Pdx1 expression in SP cells we used Pdx1-GFP knock-in mice [33]. At 1 week of age the majority of SP cells were GFP (Pdx1)-positive, a high proportion being strongly positive (Figure 5A and 5B). The proportion of Pdx1-positive SP cells decreased by weaning, but then progressively increased to plateau at 70–80% by 12 weeks of age (Figure 5A and 5B). In contrast, Pdx1-positive non-SP proportion was maximum 50% in the neonate and then dropped dramatically by weaning to remain at ∼10% (Figure 5A and 5B). This marked difference in the proportion of Pdx1-positive SP and non-SP cells was observed into adult life (Figure 5B).

Dividing cells are characterised by a higher content of RNA. Pyronin Y binds to both DNA and RNA but in the presence of Hoechst 33342 its binding is restricted to RNA. To estimate RNA content and its relationship to other properties of SP cells Pyronin Y was added for the last 15 min during Hoechst 33342 staining of pancreas cells from Pdx1-GFP mice. In SP cells from both 2 and 6 week-old mice, Pyronin Y staining correlated with Pdx1 expression (Figures 6A and 6C) and with cell size and CFP (Figure 6B and 6D). Thus, the preferential expression of Pdx1 by SP cells denotes their proliferative and colony forming potential. These findings were consistent with the small, Pdx1-negative SP cells being a less differentiated population of progenitors.

### SP Cells Proliferate, Undergo Colony Formation and Endocrine Differentiation to Insulin-secreting Cells

To investigate their differentiation potential, isolated SP cells were first allowed to proliferate and form colonies for 7–10 days. Initially, we serially diluted sorted cells down to approximately 1/well but at this level did not observe colony formation. This may reflect a requirement for more than one cell type to initiate colony formation or for cell-cell contact between the same cells. BrdU was added for the first 72 h in order to detect dividing cells. Cultures were then changed into differentiation medium for 2 weeks. Immediately after sorting and before culture, immunofluorescence staining and RT-PCR failed to reveal insulin expression by SP cells (Figure 7A and 7B). At that stage, insulin staining was detected only in mature beta cells within the non-SP. However, following culture specific proinsulin/insulin expression was detected in colony cells, including in BrdU-positive cells that had undergone division (Figure 7C). Starting with 3×10^3^ cells, we generated 20–95 colonies in which the majority of cells were insulin-positive Immunofluorescence staining and/or RT-PCR also revealed expression of glucagon and somatostatin, and the secretory protein, synaptophysin, in differentiated SP cells (Figure 7A and 7D). In response to an increase in medium glucose concentration from 1 to 20 mM, insulin secretion by SP cell-derived colonies increased by a mean of 10-fold (Figure 7E). These colonies maintained insulin expression for at least 2 weeks after transplantation into vascularised chambers of immune deficient, hyperglycaemic mice (Figure 7F), but persistent hyperglycaemia necessitated euthanasia of the mice.

## Discussion

SP cells have previously been identified in foetal human [28] and adult human [29], [30] and monkey [29] pancreas, but SP cells from the adult pancreas have not previously been shown to be endocrine progenitors. Zhang et al [28] isolated SP cells from islet-like clusters formed after culture of collagenase-digested foetal pancreas and found that in growth factor-supplemented serum-free medium they gave rise to insulin-containing cells. We characterized purified SP cells from the adult pancreas, free of any possible de-differentiated endocrine cells or hematopoietic stem cells. We aimed to demonstrate the dynamic response of SP cells to injury and their capacity to differentiate into endocrine cells capable of glucose-responsive insulin secretion.

The significance of the SP as a potential source of endocrine progenitors was shown by expansion of SP cells in response to β cell or pancreas injury. Previous studies of β cell regeneration in rodent models have not always distinguished response to injury and hyperglycaemia [12], [14], [43]. We documented SP expansion without hyperglycaemia in the partial pancreatectomy model. We then showed that, under defined conditions *in vitro*, SP cells proliferated and differentiated into endocrine tissue containing glucose-responsive insulin-secreting cells. SP-derived cells expressing insulin had undergone division, indicated by co-localisation of BrdU. Their maturation was reflected by expression of the secretory vesicle protein, synaptophysin. When transplanted into vascularised chambers of immune-deficient diabetic mice SP-derived endocrine tissue maintained insulin expression for at least 2 weeks. Although persisting hyperglycaemia necessitated euthanasia of the mice, injection of insulin-secreting cells into the vascularised chambers was tractable and their survival an indication of their potential to reverse less severe hyperglycaemia.

SP cells represented ∼ 1% of total pancreas cells in the neonate and this proportion, along with CFP, decreased 30-fold by 12 weeks of age, consistent with results from other organs [22]–[25]. This may reflect progressive utilisation of resident SP cells for tissue re-modelling in early life and may have implications for regenerating cells in later life. Indeed, this age-related decrease in pancreatic SP cells mirrors the age-related decrease in pancreas regeneration after partial pancreatectomy in the mouse [44] and Pdx1 expression in the human pancreas [45].

Because the transcription factor Pdx1 is critical for maintenance of β cell function and β cell regeneration in the adult [7], [33] we studied SP cells from a mouse in which Pdx1 expression was marked by GFP knocked into the *Pdx1* locus. The majority of CD45^−^ SP cells were Pdx1^+^ and the level of Pdx1 expression correlated with the capacity of SP cells to proliferate and form colonies. By the end of the growth-differentiation culture period the majority (>90%) of cells within colonies were Pdx-1^+^. A minority of SP cells (≤10%) were very small with a high nucleus to cytoplasm ratio and low eosin staining. These cells were Pdx1^−^ and did not form colonies. They may be more primitive, quiescent stem cells since we observed that their cytoplasm increased and they became Pdx1^+^ after culture in growth conditions (results not shown). A minor population of small cells with extremely low side scatter was also reported in ocular surface epithelia SP [45]. Together with results from the β cell injury and *in vitro* culture studies, these findings highlight the key role for Pdx1 in endocrine progenitor cells in the SP.

We could not distinguish CD45^−^ SP cells by markers that have been used to characterise stem cells in other tissues. For example, expression of c-kit**^+^** (hematopoietic) and Thy-1**^+^** (mesenchymal) was similar on SP and non-SP cells, except for a small number of Thy-1^high^ non-SP cells. c-kit and Thy-1 have also been detected on SP cells from other tissues [24] but comparisons with non-SP cells are generally lacking. Expression of the incretin-degrading enzyme CD26 and adhesion molecules CD29, CD49f and CD24 was similar on SP and non-SP cells, as reported for mouse skin [25] and mammary gland [47]. The majority of pancreas SP cells did not express Sca-1, also reported for SP cells from other tissues [24], but again without data for non-SP cells. Sca-1 was not a marker of endocrine progenitor cells because Sca-1^+^ SP cells did not express Pdx1. Expression of the pancreatic duct markers, CD133, CD326 and DBA lectin, reported on some stem/progenitor cells, was similar on SP and non-SP cells. Apart from Pdx1, the only marker that enriched for CFP within the CD45^−^ SP population was CD326, expressed on the majority of SP cells, but only a minority of non-SP cells. CD326 is required for intercellular adhesion of pancreatic epithelial cells during development and maintenance of stem cell phenotype [40], [41].

The regenerative capacity of SP cells has previously been demonstrated in several adult, non-pancreatic tissues [24]. For example, bone marrow SP cells reconstituted haematopoiesis in lethally irradiated mice [22], mammary gland SP cells formed mammary outgrowths when transplanted into cleared fat pads [46], liver SP cells generated mature hepatocytes and bile duct epithelium in livers of mice treated with 3,5-diethoxycarbonyl-1,4-dihydrocollidine (DDC) [48] and muscle SP cells formed dystrophin-positive muscle fibers in *mdx* mice [49]. Our findings demonstrate that the SP in the adult mouse pancreas is a dynamic reservoir of progenitor cells, including cells that give rise to insulin-secreting cells. SP cells may therefore have the potential to regenerate or replace lost β cells in diabetes.
